# Estrogen receptor β exerts tumor suppressive effects in prostate cancer through repression of androgen receptor activity

**DOI:** 10.1371/journal.pone.0226057

**Published:** 2020-05-15

**Authors:** Surendra Chaurasiya, Scott Widmann, Cindy Botero, Chin-Yo Lin, Jan-Åke Gustafsson, Anders M. Strom

**Affiliations:** 1 Department of Biology and Biochemistry, University of Houston, Center for Nuclear Receptors and Cell Signaling, Science & Engineering Research Center, Houston, Texas, United States of America; 2 Department of BioSciences and Nutrition, Karolinska Institutet, Novum, Huddinge, Sweden; Universita degli Studi della Campania Luigi Vanvitelli, ITALY

## Abstract

Estrogen receptor β (ERβ) was first identified in the rodent prostate and is abundantly expressed in human and rodent prostate epithelium, stroma, immune cells and endothelium of the blood vessels. In the prostates of mice with inactivated ERβ, mutant phenotypes include epithelial hyperplasia and increased expression of androgen receptor (AR)-regulated genes, most of which are also upregulated in prostate cancer (PCa). ERβ is expressed in both basal and luminal cells in the prostate while AR is expressed in luminal but not in the basal cell layer which harbors the prostate stem cells. To investigate the mechanisms of action of ERβ and its potential cross-talk with AR, we used RNA-seq to study the effects of estradiol or the synthetic ligand, LY3201, in AR-positive LNCaP PCa cells which had been engineered to express ERβ. Transcriptomic analysis indicated relatively few changes in gene expression with ERβ overexpression, but robust responses following ligand treatments. There is significant overlap of responsive genes between the two ligands, estradiol and LY3201 as well as ligand-specific alterations. Gene set analysis of down-regulated genes identified an enrichment of androgen-responsive genes, such as FKBP5, CAMKK2, and TBC1D4. Consistently, AR transcript, protein levels, and transcriptional activity were down-regulated following ERβ activation. In agreement with this, we find that the phosphorylation of the CAMKK2 target, AMPK, was repressed by ligand-activated ERβ. These findings suggest that ERβ-mediated signaling pathways are involved in the negative regulation of AR expression and activity, thus supporting a tumor suppressive role for ERβ in PCa.

## Introduction

The prostate is an androgen-responsive organ which is controlled by the androgen receptor (AR) both under normal physiological conditions and in malignancy. AR plays an important role in PCa as a strong driver of proliferation, and as such is the primary target for treatment of PCa [1]. Recently it was shown that androgen signaling is essential for PCa tumorigenesis from prostatic basal cells[2]. In addition to the 2.5 million people living with PCa, 250,000 new cases are diagnosed with 28,000 men dying from PCa every year in the United States alone. Treatment strategies differ depending on factors such as the stage of the disease, the age and physical status of the patient, and what treatments patients have previously received. PCa is a very indolent cancer and can initially be treated with surgery or androgen deprivation therapy (ADT), however, ADT often leads to emergence of a more aggressive castrate resistant metastatic cancer (CRPCa) [3]. Prostate cancer frequently has changed metabolism and in accordance with this CAMKK2 has been found to be a target of AR which affects PCa cell growth, migration and survival. In addition, this protein kinase affects bone remodeling and macrophage function, and is a candidate target downstream of AR for controlling aggressive PCa and preventing ADT-induced bone loss[4].

In addition to AR, the prostate also expresses estrogen receptor β (ERβ), which is expressed in both basal and luminal cells [5]. ERβ, a member of the nuclear receptor family, was discovered in 1996 [6]. This receptor has been shown to act as a tumor suppressor in several types of cancer [7–9]. In addition, when ERβ is deleted from the mouse genome, epithelial hyperplasia and upregulation of AR-regulated genes in the prostate occur[10]. ERβ is ligand-activated, which like ERα, can be activated by estradiol. However, in the prostate the more abundant ERβ ligand is not 17β-estradiol, but 17β-Adiol [11]. The basal cells in the prostate which are thought to harbor the prostatic stem cells, are AR-negative. Deletion of ERβ in the prostate gives rise to hyper proliferation of the epithelial layer and increases the proportion of intermediary luminal cells (luminal cells which are not fully differentiated) [10]. ERβ has previously been shown to reduce AR activity in the prostate by increasing the expression of the co-repressor DACH1/2 [12] and decreasing the AR driver RORc. In addition to DACH1/2, the co-repressor NCoR2/SMRT has also been shown to affect the activity of AR [13]. The overall effect of ERβ on gene networks in PCa cells has not been reported. The present study describes the first transcriptomic analysis of ERβ activation in AR-positive PCa cells and reveals a key role for ERβ in regulating AR expression and activity in PCa.

## Materials and methods

### Reagents and cell culture

The LNCaP and 22Rv1 cell lines were obtained from the American Type Culture Collection (ATCC). LNCaP and 22RV1 cells were maintained in RPMI-1640 (Invitrogen Inc., Carlsbad, CA) medium supplemented with 10% fetal bovine serum (FBS) (Sigma, St. Louis, MO), and Antibiotic-Antimycotic (Invitrogen Inc., Carlsbad, CA). All experiments used cells below passage 30. Compounds and concentrations used was R1881 (1 nM), 17β-estradiol (10 nM), (Sigma St. Louis, MO), and LY3201 (10 nM) which was a gift from Elli Lilly Indianapolis, IN. All treatments were for 24 hours.

### LNCaP cell lines expressing control virus or ERβ containing virus

LNCaP cells were infected with the lentivirus Lenti6-TOPO-V5-D, empty or containing cDNA for human ERβ at 2 m.o.i (multiples of infection). The lentivirus vector was obtained from Invitrogen Carlsbad, CA and the cloning of ERβ cDNA is described in Williams et al. [14]. The control in all experiments is cells infected with empty virus vector.

### Protein extract preparation

Whole-cell extracts, were prepared by washing cells twice with PBS, lysed in 2 times packed cell volume of lysis buffer [RIPA buffer supplemented with 1 mM dithiothreitol, 1 mM phenylmethylsulfonyl fluoride, protease inhibitor cocktail, PhosStop (Roche, Indianapolis, IN)] for 15 minutes on ice and then centrifuged at 14,000 x g for 10 minutes.

### Western blotting

Thirty micrograms of protein were loaded on an SDS-PAGE 10% Bis-Tris gel with Tris running buffer and transferred to a PVD membrane after electrophoretic separation. Membranes were blocked with 5% non-fat powdered milk in 0.1% TBST buffer and probed with anti-AR (sc-816), GAPDH-HRP (sc-47724), AMPK (2532S), pAMPK 2535S, FKBP5 122105 (Cell Signaling Technology Danvers, MA), CAMKK2 (H00010645-M01) (Abnova Taipei, Taiwan), β-Actin (A1978) (Sigma Millipore Sigma, St. Louis, MO). Primary antibodies were used at 1:200–1000 dilutions, and secondary antibody was used at 1:10,000. The western blot experiments were minimally repeated two times.

### Proliferation assay

Control and ERβ LNCaP cells were grown with blasticidin for 48 hrs in complete medium till subconfluency. Cells were trypsinized, washed with PBS, counted and suspended in 10% DCC-FBS. In a 96-well plate, 5000 cells/ well were added along with the indicated ligand and incubated for 5 days at 37C in 5% CO_2_ and 95% humidity. On the day of assay 10 μl MTS was reagent added to each well and incubated at 37C ° for 60 min and the color was measured at 490nM with SpectraMax spectrophotometer.

### RNA extraction and real-time PCR

RNA extraction was performed with Qiagen mRNA extraction kit according to standard protocol. cDNA was synthesized from 1μg of total RNA with First Strand System according to standard protocol (Invitrogen Inc. NY). Real-time PCR was performed with SYBR Green iTaq master mix (BioRad Hercules, CA). Primers (Integrated DNA Technologies, Inc. Coralville, IA) were: GAPDH; F, 5’-TGACAACTTTGGTATCGTGGAAGG-3’ and R, 5-AGGCAGGGATGATGTTCTGGAGAG-3 (reference gene); AR; F, 5’-TCACCAAGCTCCTGGACTCC-3’ R, 5’-CGCTCACCATGTGTGACTTGA 3’; FKBP5; F, 5’- ATTGGAGCAGGCTGCCATTGTC -3’, R, 5’-CCGCATGTATTTGCCTCCCTTG-3’, CAMKK2; F, 5’-TCCAGACCAGCCCCGACATAG-3’, R, 5’-CAGGGGTGCAGCTTGATTTC-3’ 7500 Fast Real-Time PCR System (Applied Biosystems) using optimized conditions for SYBRGreen I dye system: 50 C for 2 minutes, 95 C for 10 minutes, followed by 40–50 cycles at 95 C for 15 seconds and 60 C for 50 seconds. Optimum primer concentration was determined in preliminary experiments, and amplification specificity confirmed by dissociation curve. The variance between the groups that are being statistically compared is similar.

### RNA-sequencing

Poly(A) mRNA was isolated using NEBNext poly(A) mRNA Magnetic Isolation Module. Libraries were prepared using NEBNext Ultra II RNA Library Prep Kit for Illumina. Sequencing was performed on NovaSeq 6000 with 150 bp paired-end reads. Treatments include three independent replicates.

### Transcriptome analysis

Reads were aligned to reference genome (GRCh38) indexes using STAR[15] (v2.5). HTSeq[16] (v0.6.1) was used for mapped gene count quantification. Differential expression analysis was performed using DESeq2[17] (1.24.0). The resulting P-values were adjusted using the Benjamini and Hochberg’s method. Genes with an adjusted P-value <0.05 found by DESeq2 were assigned as differentially expressed. Venn diagrams and heat map were prepared in R. Gene set enrichment analysis (GSEA)[18, 19] was performed using rankings based on the test statistic from differential expression analysis and the hallmarks gene set (h.all.v7.0.symbols.gmt).

Gene Ontology (GO) enrichment analysis of differentially expressed genes was implemented by the cluster Profiler R package, in which gene length bias was corrected. GO terms with corrected P-values less than 0.05 were considered significantly enriched by differentially expressed genes. The RNA-seq data is available in NCBI’s Gene Expression Omnibus through accession number GSE144800.

## Results

### The transcriptomic effects of ERβ in the AR-positive cell line LNCaP

**Fig 1:**
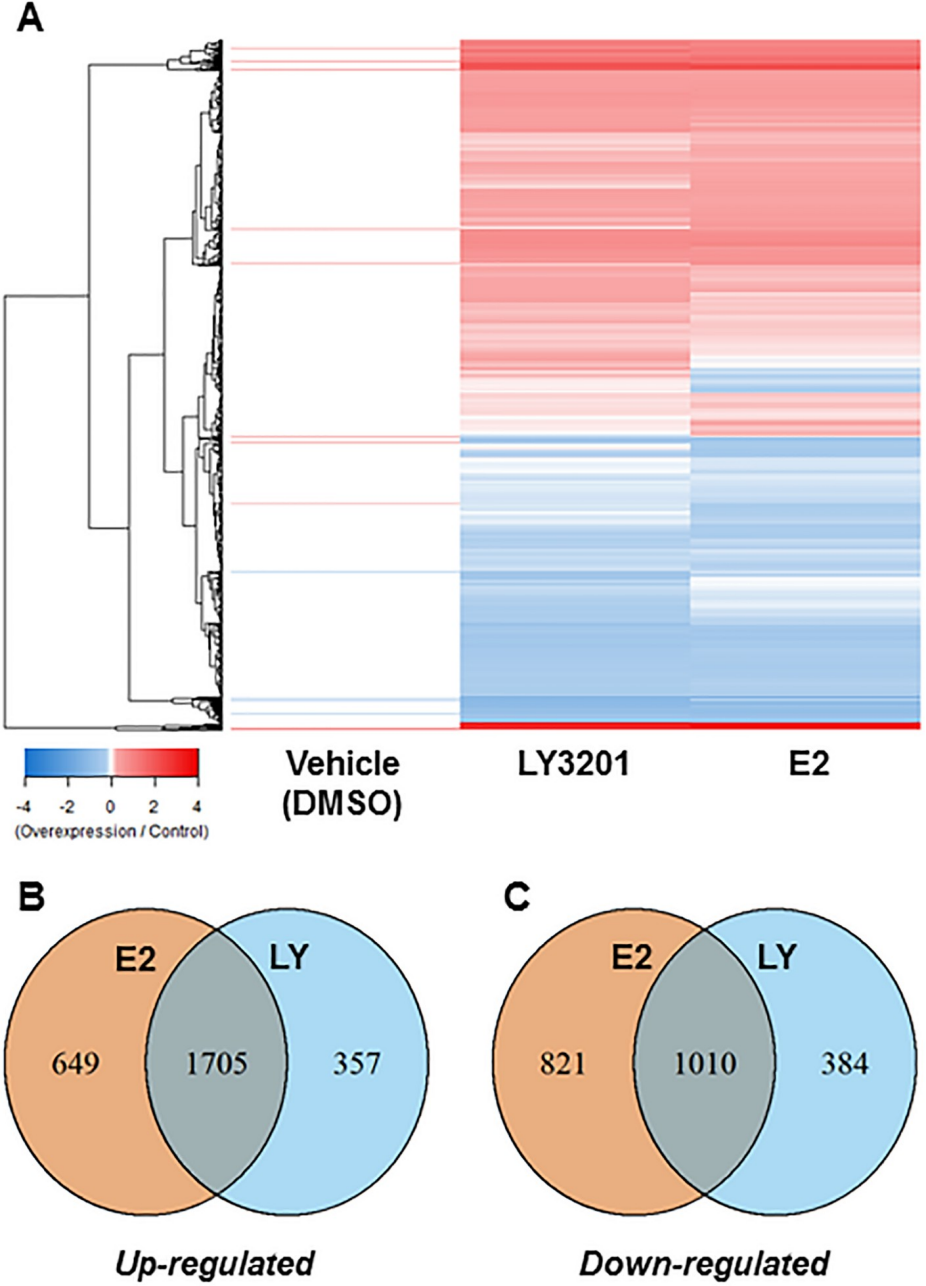
RNAseq analysis identified differentially expressed genes which respond to ERβ activation. A, Gene expression profiles of responsive genes in cells treated with vehicle, E2, or LY3021 are presented in a heatmap of log_2_-transformed fold-change values. Hierarchical clustering of expression profiles and resulting dendrogram group genes based on their similarities in responses to ligand treatment. B, Venn diagrams show the proportion of responsive genes which are common and specific to each of the two ERβ ligands used in the study.

To determine the functions of ERβ in AR-positive PCa, we used RNA-seq to compare the transcriptomes of ERβ over-expressing and non-expressing LNCAP cells treated with vehicle (DMSO), and ERβ ligands estradiol (E2) and LY3201. Differential expression analysis consisted of comparing over- vs non-expressing ERβ cells within each treatment regime. DMSO-treatment had very little effect on gene expression (Fig 1A (heat map)). Comparison of responsive genes to previously published datasets obtained in other cancer cell lines indicates cell-type specific responses with relatively few overlaps in response between cell lines from different cancer types (S1 Fig) [20–22]. E2 and LY3201 treatments elicited changes in 4185 and 3456 differentially expressed genes (adjusted p-value < 0.05), respectively. The proportion of upregulated (60%) and downregulated (40%) genes between the two ligands was similar (Table 1). With the upregulated genes there was a strong overlap between the two treatments (63%). However, there was only a modest overlap in the downregulated genes shown (46%). Ligand-dependent differentially expressed genes were less prevalent in LY3201 treated cells (Fig 1B).

**Table 1:** Summary of numbers of differentially expressed genes identified in RNAseq study.

	Differentially Expressed Genes*		
Treatment	*Up-regulated*	*Down-regulated*	*Total*
Vehicle (DMSO)	29	14	43
E2	2354	1831	4185
LY	2062	1394	3456

*Defined as those with FDR-adjusted p<0.05.

**Fig 2:**
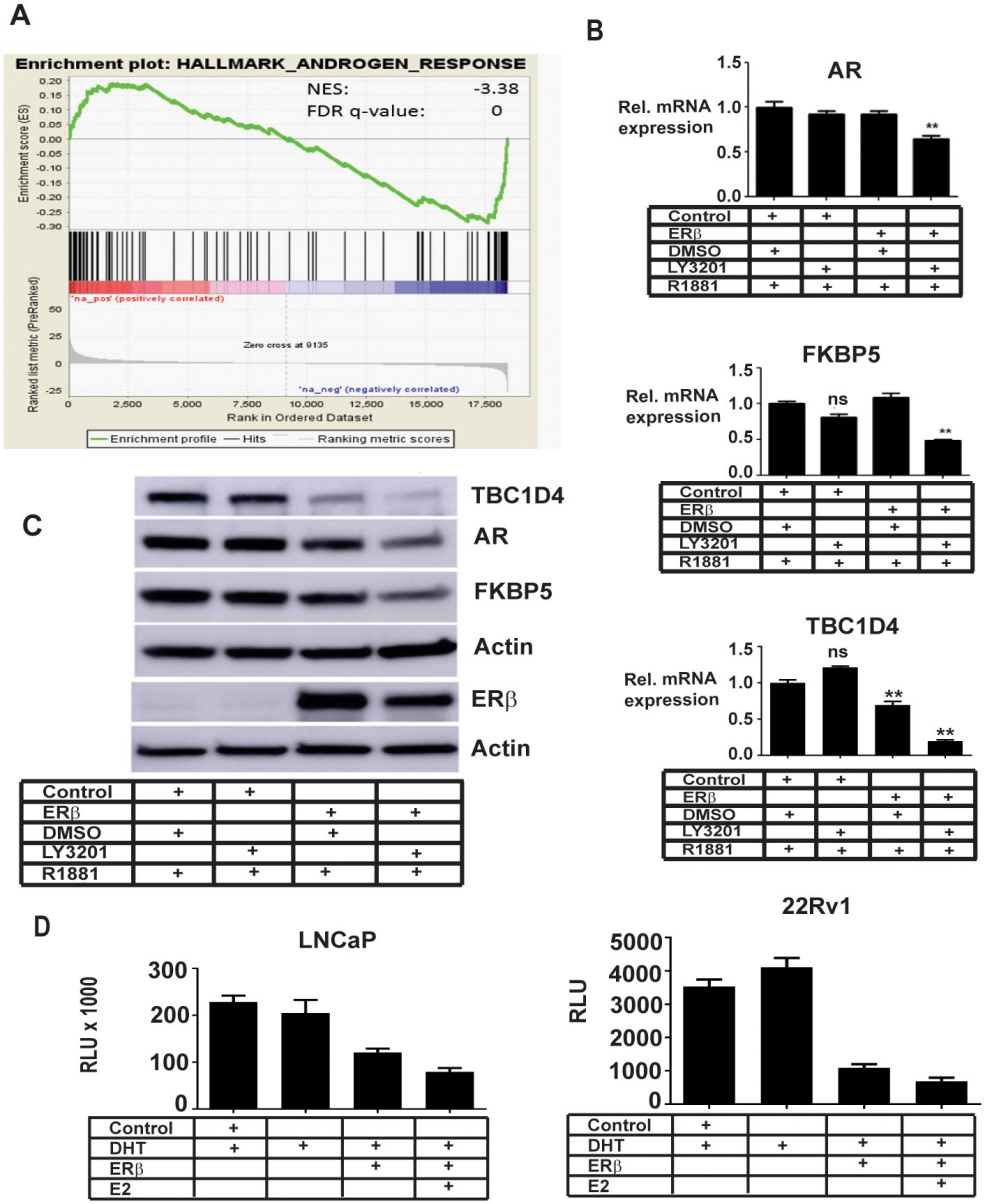
ERβ activation inhibits AR expression and transcriptional activity. A, Gene Set Enrichment Analysis (GSEA) revealed an enrichment of genes involved in androgen response down-regulated by ERβ activation (negative enrichment (NES) score). B, Transcript (graphs) and C, protein (western images) levels of AR and AR target genes TBC1D4 and FKBP5 were reduced by ERβ expression and treatment with LY3021. D, AR transcriptional activity was reduced by ERβ expression and activation in reporter gene assays in AR-positive LNCaP and 22Rv1 prostate cancer cells.

Pre-ranked gene set enrichment analysis (GSEA) was used to identify pathways and mechanisms relevant to ERβ expression and activation in PCa. We examined those genes which are commonly regulated by LY3201 and E2 for enrichment analysis. The most positively enriched hallmark gene set in ligand-treated cells were genes responsive to estrogen; other highly enriched sets contained genes involved in MTORC1 signaling and the unfolded protein response. GSEA also revealed downregulated genes involved in several pathways associated with cancer hallmarks such as hypoxia and glycolysis (Table 2). The most negatively enriched set contained genes involved in response to androgen, a central driver in androgen-sensitive PCA (see Fig 2A). Additional enrichment analysis using other gene set categories (KEGG and Reactome) are found in supplementary S2 Table. These androgen-responsive genes were investigated further due to the well-established importance of androgen signaling in prostate cancer. Further comparison of AR-regulated genes from other published datasets with the expression data from this study also indicated a negative correlation with their responses following ERβ activation (S2 Fig) [23, 24]. For full list of regulated genes see S1 Table.

**Table 2:** Summary of top hits from Gene Set Enrichment Analysis (GSEA) of ERβ ligand-responsive differentially expressed genes commonly regulated by E2 and LY treatments identified in RNAseq study.

Gene Set	NES	FDR q-value*
*Positive Enrichment Score*		
HALLMARK_ESTROGEN_RESPONSE_EARLY	5.494334	0
HALLMARK_ESTROGEN_RESPONSE_LATE	4.332718	0
HALLMARK_UNFOLDED_PROTEIN_RESPONSE	2.781836	0
HALLMARK_COAGULATION	2.368938	0.003125
HALLMARK_UV_RESPONSE_UP	2.265393	0.006134
HALLMARK_PEROXISOME	2.030336	0.017496
HALLMARK_XENOBIOTIC_METABOLISM	1.807741	0.062153
HALLMARK_MITOTIC_SPINDLE	1.717	0.088724
HALLMARK_TNFA_SIGNALING_VIA_NFKB	1.643714	0.114817
HALLMARK_INTERFERON_GAMMA_RESPONSE	1.624198	0.113491
*Negative Enrichment Score*		
HALLMARK_ANDROGEN_RESPONSE	-2.35927	0.003696
HALLMARK_UV_RESPONSE_DN	-1.63909	0.174314
HALLMARK_SPERMATOGENESIS	-1.46447	0.244085
HALLMARK_KRAS_SIGNALING_DN	-1.14242	0.633201
HALLMARK_G2M_CHECKPOINT	-1.10358	0.573174
HALLMARK_P53_PATHWAY	-0.97801	0.701626
HALLMARK_TGF_BETA_SIGNALING	-0.93594	0.672351
HALLMARK_IL2_STAT5_SIGNALING	-0.85269	0.716611

*FDR q<0.05

Table 1 shows differentially expressed genes up-regulated and down-regulated after treatment with E2 or LY3201

Table 2 shows hallmarks from Gene Set Enrichment Analysis (GSEA) positive and negative enrichment scores. NES Normalized Enrichment Score and FDR q-value False Discovery Rate.

### ERβ reduces the AR activity in LNCaP cells

Since AR-responsive genes were affected by ERβ activation (see Fig 2A), we investigated whether expression and/ or activity of AR was changed by expression of ERβ as has been proposed by several studies based on ERβ function in the mouse [11, 12]. We found that AR, FKBP5 and TBC1D4 mRNA were down-regulated by both estradiol and LY3201 in ERβ-expressing LNCaP cells (see Fig 2B). In addition, the protein level of the established AR-regulated genes FKBP5 and the CAMKK2 regulated gene TBC1D4 was found to be down-regulated by ERβ. (see Fig 2C). Other established AR targets *(NDRG1, B2M, SORD, TPD52, DHCR24, ADAMTS1, NKX3A, RAB4, ANKH, TSC22, MAF [24]) were similarly down-regulated by ERβ ligand treatments according to the RNA-seq data (See Table 3).

**Table 3:** List of androgen-responsive genes differentially regulated by ligand-activated ERβ in prostate cancer cells.

*Up-regulated*			*Down-regulated*		
Symbol	log_2_ FC	p-value*	Symbol	log_2_ FC	p-value*
FADS1	2.4	0	AKAP12	-1.7	1.6E-18
KRT19	1.9	1.8E-57	STEAP4	-1.2	7.8E-05
HOMER2	1.3	2.6E-80	MAF	-1.2	1.3E-57
SCD	1.0	2.2E-97	ADAMTS1	-1.1	6.6E-42
TARP	0.9	2.0E-08	CAMKK2	-1.0	3.6E-80
ACTN1	0.9	2.3E-73	NDRG1	-0.9	1.2E-09
CENPN	0.8	1.6E-33	PTPN21	-0.9	3.0E-28
ALDH1A3	0.7	7.2E-20	ZMIZ1	-0.8	3.0E-30
ELOVL5	0.7	5.2E-32	PMEPA1	-0.8	5.0E-48
CCND1	0.6	3.6E-29	ELK4	-0.8	2.7E-35
B4GALT1	0.6	6.6E-23	HERC3	-0.7	0.002
SPCS3	0.5	4.4E-13	ARID5B	-0.7	1.6E-24
SLC38A2	0.4	7.8E-14	UAP1	-0.7	3.1E-41
DBI	0.4	8.8E-08	FKBP5	-0.7	6.4E-06
H1F0	0.4	2.0E-12	IQGAP2	-0.7	1.9E-15
ABHD2	0.4	1.2E-14	RAB4A	-0.6	1.4E-23
ELL2	0.4	5.6E-06	ZBTB10	-0.6	1.7E-19
INSIG1	0.3	7.1E-08	ABCC4	-0.5	3.3E-18
HMGCS1	0.3	8.1E-11	ACSL3	-0.5	6.5E-11
LMAN1	0.3	6.1E-10	APPBP2	-0.5	5.7E-16
MYL12A	0.3	6.8E-08	TNFAIP8	-0.5	8.1E-06
SEC24D	0.3	1.3E-05	STK39	-0.5	1.6E-11
IDI1	0.2	0.0001	CDC14B	-0.4	0.001
SRF	0.2	0.003	NKX3-1	-0.4	4.1E-15
TMEM50A	0.2	0.02	INPP4B	-0.4	7.3E-05
KLK3	0.2	0.0002	TSC22D1	-0.4	5.0E-10
RRP12	0.2	0.005	TPD52	-0.4	1.4E-05
PDLIM5	0.2	0.009	SPDEF	-0.4	4.5E-07
PTK2B	0.2	0.03	HMGCR	-0.3	2.8E-09
NCOA4	0.2	0.006	TMPRSS2	-0.3	2.0E-05
			SLC26A2	-0.2	0.001
			DHCR24	-0.2	3.4E-05
			PIAS1	-0.2	0.05
			MAP7	-0.2	0.04
			UBE2J1	-0.2	0.01
			ANKH	-0.1	0.05

*FDR adjusted p≤0.05.

Table 3: Known AR regulated genes up or down-regulated in LNCaP cells expressing ERβ and treated with R1881 and LY3201 compared to control LNCaP cells treated with R1881 and LY3201.

### ERβ reduces activation of the p (ARR) _2_ PB-LUC reporter in LNCaP and 22Rv1 cells and reduces AMPK phosphorylation

We used AR-reporter constructs to investigate whether AR activity is directly affected by ERβ in ERβ-overexpressing LNCaP cells as well as 22Rv1 PCa cells. Ligand-treatment of transfected cells showed ERβ-dependent inhibition of AR-reporter activity compared to control transfected cells (see Fig 2D for luciferase assay).

### ERβ-regulation of CAMKK2 and its downstream targets

**Fig 3:**
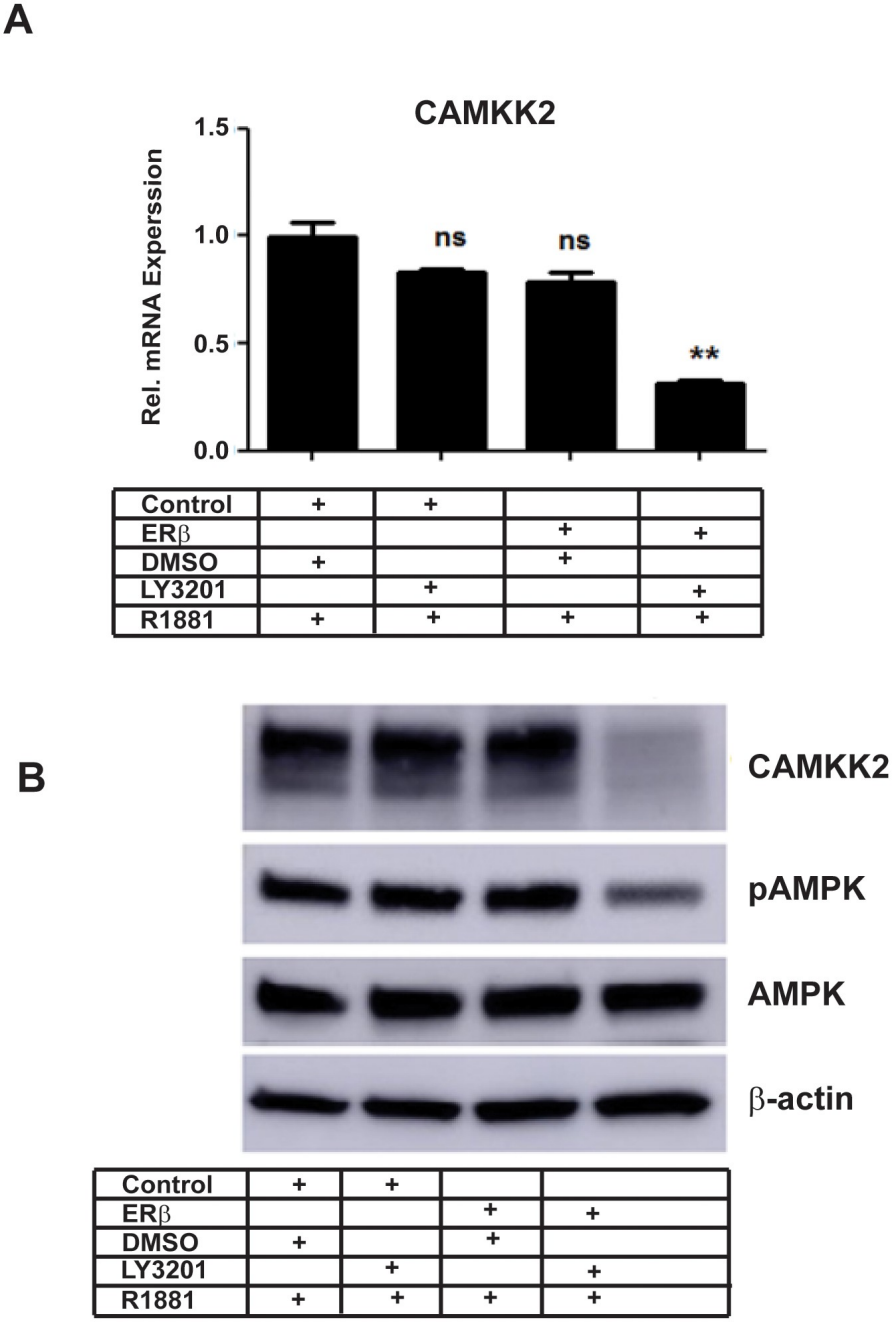
Expression and activity of AR target gene CAMKK2 are disrupted by ERβ. A, AR target gene CAMKK2 transcript levels were reduced by ERβ activation. B, CAMKK2 protein expression and activity (measured by anti-AMPK and anti-phospho-AMPK antibodies raised against the CAMKK2 substrate) were down-regulated by ERβ.

We decided to focus on the AR-regulated gene, CAMKK2, which is a promising downstream target of AR and has been shown previously to affect PCa survival, metabolism, cell growth, and migration [4], It was repressed by ligand-activated ERβ (see Fig 3A). To determine whether events downstream of CAMKK2 were affected by the presence of activated ERβ, we analyzed the activity of AMPK by measuring its phosphorylated form (pAMPK). There was a clear reduction in pAMPK following treatment with LY3201 (Fig 3B).

### Agonist activated ERβ inhibits proliferation of LNCaP cells

**Fig 4:**
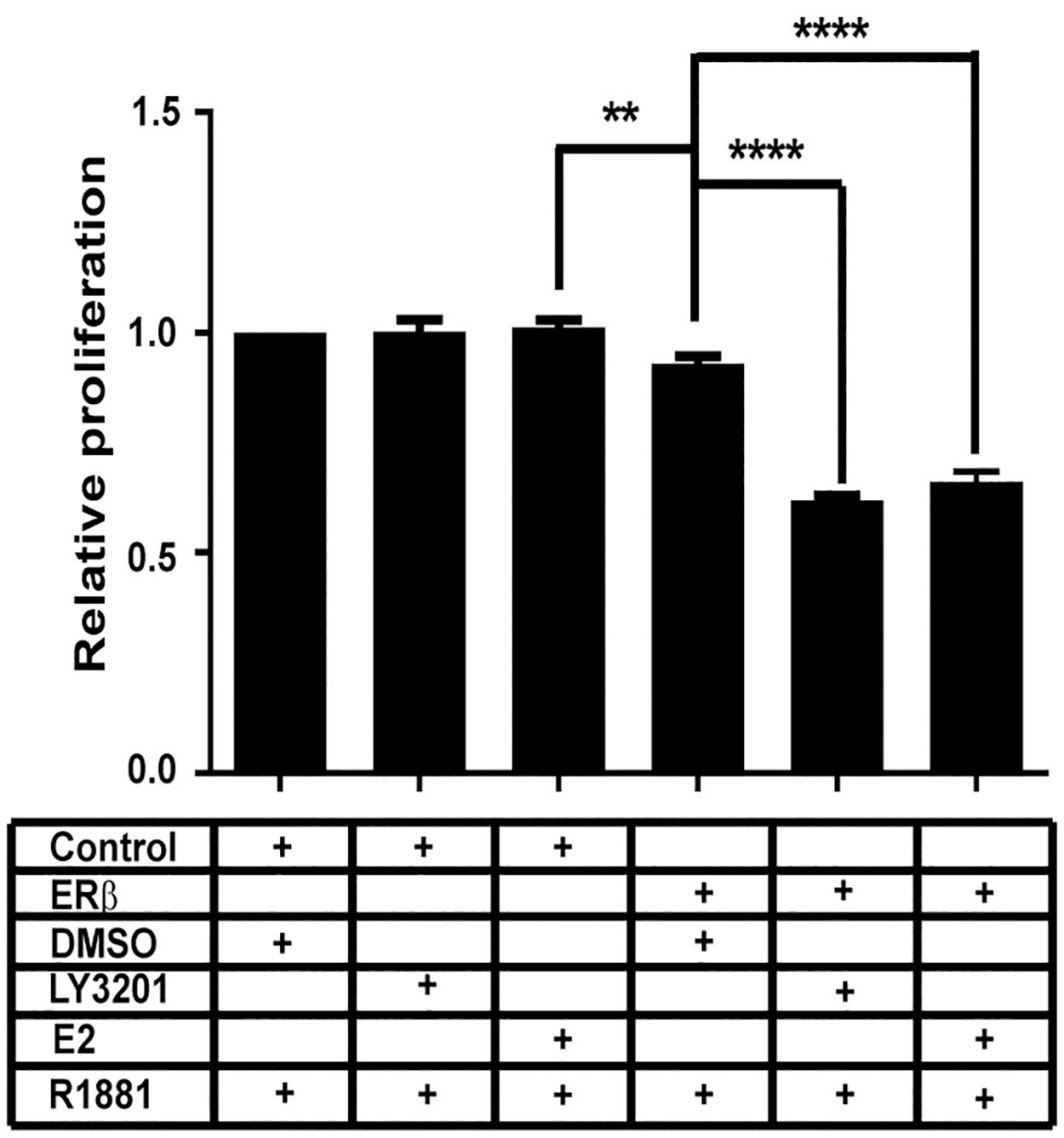
Expression of ERβ in LNCaP cells inhibits proliferation. LNCaP cells 5000 cells / well into 96 well plate were growing for 48 hours supplemented with 10nM R1881 in all wells. DMSO, LY3201 (1 μM) and E2 (10nM) in 6 wells each.

We analysed ERβ expressing LNCaP cells for changes in cell growth. In agreement with the RNA seq data we see the inhibition of proliferation only in the presence of agonists either LY3201 or E2 (Fig 4).

## Discussion

In the prostate of ERβ knockout mice, there is an increase in the number of basal cells (p63-positive) and poorly differentiated intermediary cells [10] as well as a decrease in fully differentiated luminal cells[11, 12]. ERβ is expressed in both luminal (AR-positive) and basal (AR-negative) cells. From previous studies we have found that ERβ has an anti-proliferative effect in PCa by down regulating Skp2 and up-regulating p27 KIP1 protein [25].

In the present study, we specifically examined the functions and potential mechanisms of action of ERβ in AR-positive LNCaP cells by RNA-seq. Consistent with its function as a ligand-dependent transcription factor, overexpression of ERβ elicited relatively small changes in the LNCaP transcriptome in a ligand-independent fashion, but treatments with ER ligands E2 or ERβ-specific ligand LY3201 resulted in altered expression of thousands of genes. As expected, GSEA results revealed significant enrichment of ER-regulated genes among those up-regulated by ligand treatment. Of particular interest is the down-regulation of genes involved in androgen response. GSEA results and follow-up studies on the expression and activity of AR and target genes shown herein provide evidence that the tumor suppressor actions of ERβ in PCa are due to its negative regulation of AR signaling. AR expression and/or activity is often increased in primary PCa, as well as in metastatic PCa. In the present study we found that in LNCAP cells overexpressing ERβ, AR activity is repressed by ERβ-ligands, as were the classical AR targets c-Myc, FKBP5 and CAMKK2. TBC1D4 is the most down-regulated AR-responsive gene and a major regulator of glucose uptake in the prostate by inducing membrane localization of Glut12. The two most up-regulated AR responsive genes FADS1 and KRT19 are likely to be targets of both AR and ERβ, where FADS1 has been shown to be regulated by estrogen [26], and KRT19 expression correlating to estrogen receptor expressing breast cancer [27]. Not only AR has a differentiating effect in the prostate but ERβ is also differentiating, and is needed for full differentiation of luminal cells [10]. An example is cytokeratin 19, a luminal differentiation marker, which we found to be upregulated by ERβ in our RNA-seq. analysis. Mak et. al (2013) [28] showed that ERβ maintains epithelial differentiation by regulating prolyl hydroxylase 2 transcription.

We performed ChIP-seq in prostate cells but did not find ERβ binding around or within the AR gene, indicating that the regulation could be either on mRNA stability or protein stability of AR. A study by Nanni et al. showed that endothelial NOS, estrogen receptor β, and HIFs cooperate in the activation of a negative prognostic transcriptional pattern in aggressive human prostate cancer [29]. We have shown that the splice variants of ERβ, the ERβ2 and ERβ5 isoforms, interact with HIF-1α and HIF-2α while we could not detect any HIF interaction with ERβ1 (the wt form) in prostate cancer cells. Furthermore, we have shown that the splice variants are oncogenic [30, 31]. The opposite effects of the wt ERβ and the variants on prostate cancer requires further studies.

The repression of AR by ERβ is not limited to LNCaP cells, but also occurs in another androgen-responsive cell line, 22Rv1, which expresses both normal AR as well as the AR variant AR7. In addition to regulating AR, GSEA and additional gene ontology analysis of genes differentially expressed following ERβ activation revealed an enrichment of genes involved in cancer-related processes, including apoptosis, response to hypoxia, KRAS signaling, and key metabolic pathways. As in many other studies we observe that expression of ERβ causes inhibition of proliferation [7, 9, 25]. These findings reveal that re-expression and activation of ERβ can suppress oncogenic mechanisms in androgen-responsive cancer cells.

## Supporting information

S1 Fig Comparison of differentially expressed genes identified in this study to similar datasets generated in cell lines derived from other types of cancers. (TIF)

S2 Fig Comparison of differentially expressed genes identified in this study to AR target/regulated genes identified in other studies showed negative correlation between ERbeta regulation and AR regulation. (TIF)

S1 Table Differential gene expression. DMSO control Vs. LY ERβ Overexpression.
(XLSX)

S2 Table Top GSEA categories for DEGs common between LY and E2 treatments. LY values, Hallmarks gene sets.
(XLSX)

S1 Raw images (PDF)
