# Supplementary figures and images for: Estrogen receptor β exerts tumor suppressive effects in prostate cancer through repression of androgen receptor activity
Source: PLoS One. 2020 May 15;15(5):e0226057. doi: 10.1371/journal.pone.0226057 (PMC7228066; doi:10.1371/journal.pone.0226057)

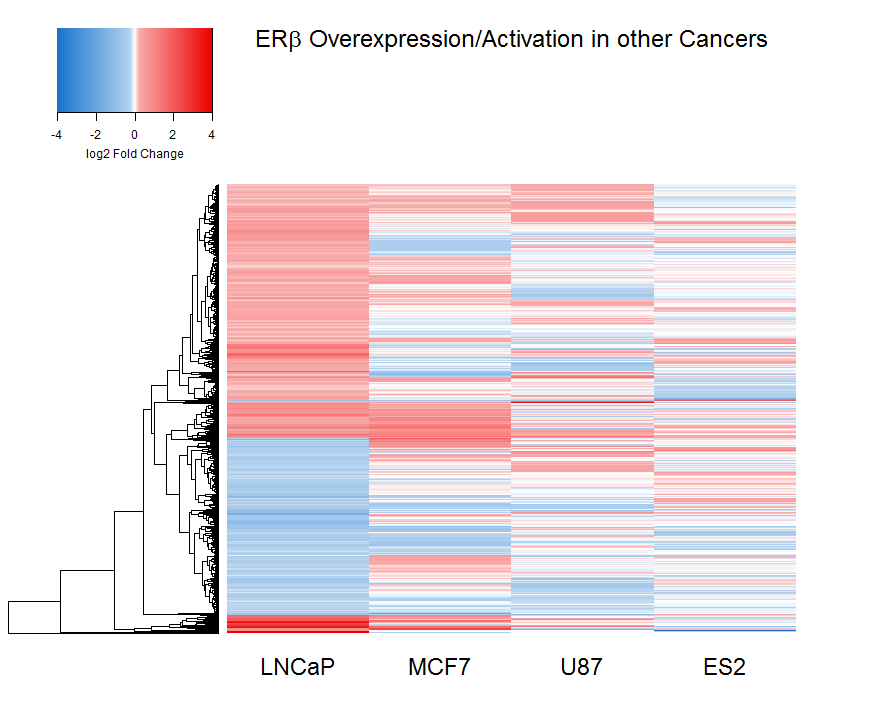

Supplement: S1 Fig — (TIF) [file pone.0226057.s001.tif]

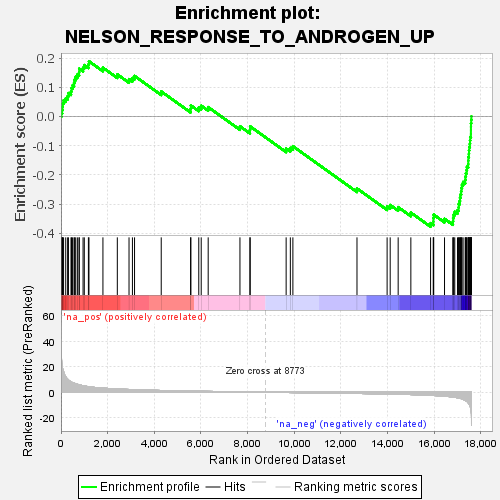

Supplement: S2 Fig — (TIF) [file pone.0226057.s002.tif]
