# Supplementary material for: Estrogen receptor β exerts tumor suppressive effects in prostate cancer through repression of androgen receptor activity
Source: PLoS One. 2020 May 15;15(5):e0226057. doi: 10.1371/journal.pone.0226057 (PMC7228066; doi:10.1371/journal.pone.0226057)
Supplement: S1 Raw images — (PDF) [file pone.0226057.s005.pdf]

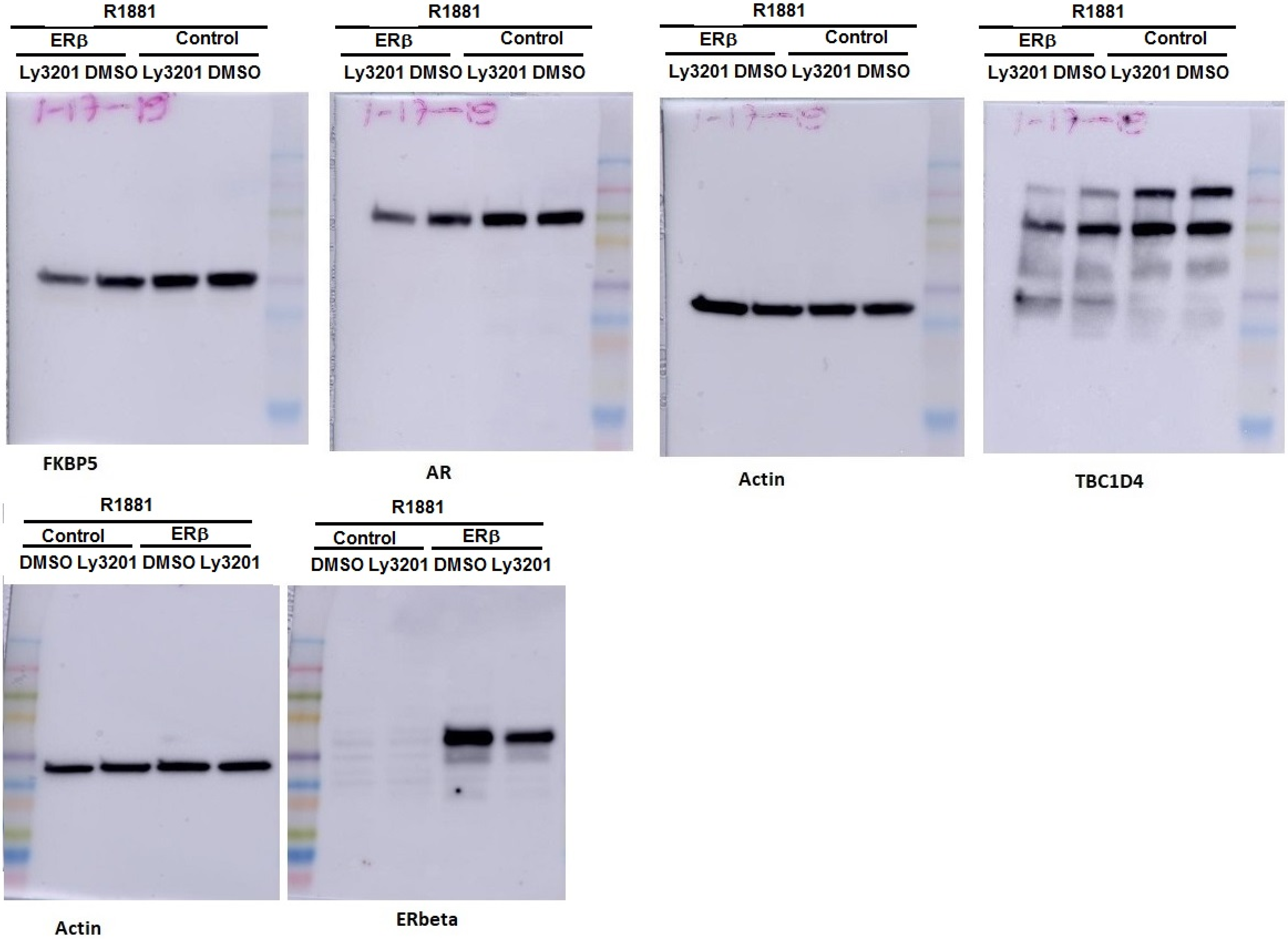
R1881
ERβ
Control
Ly3201 DMSO Ly3201 DMSO
FKBP5
R1881
ERβ
Control
Ly3201 DMSO Ly3201 DMSO
AR
R1881
ERβ
Control
Ly3201 DMSO Ly3201 DMSO
Actin
R1881
ERβ
Control
Ly3201 DMSO Ly3201 DMSO
TBC1D4
R1881
Control
ERβ
DMSO Ly3201 DMSO Ly3201
Actin
R1881
Control
ERβ
DMSO Ly3201 DMSO Ly3201
ERbeta

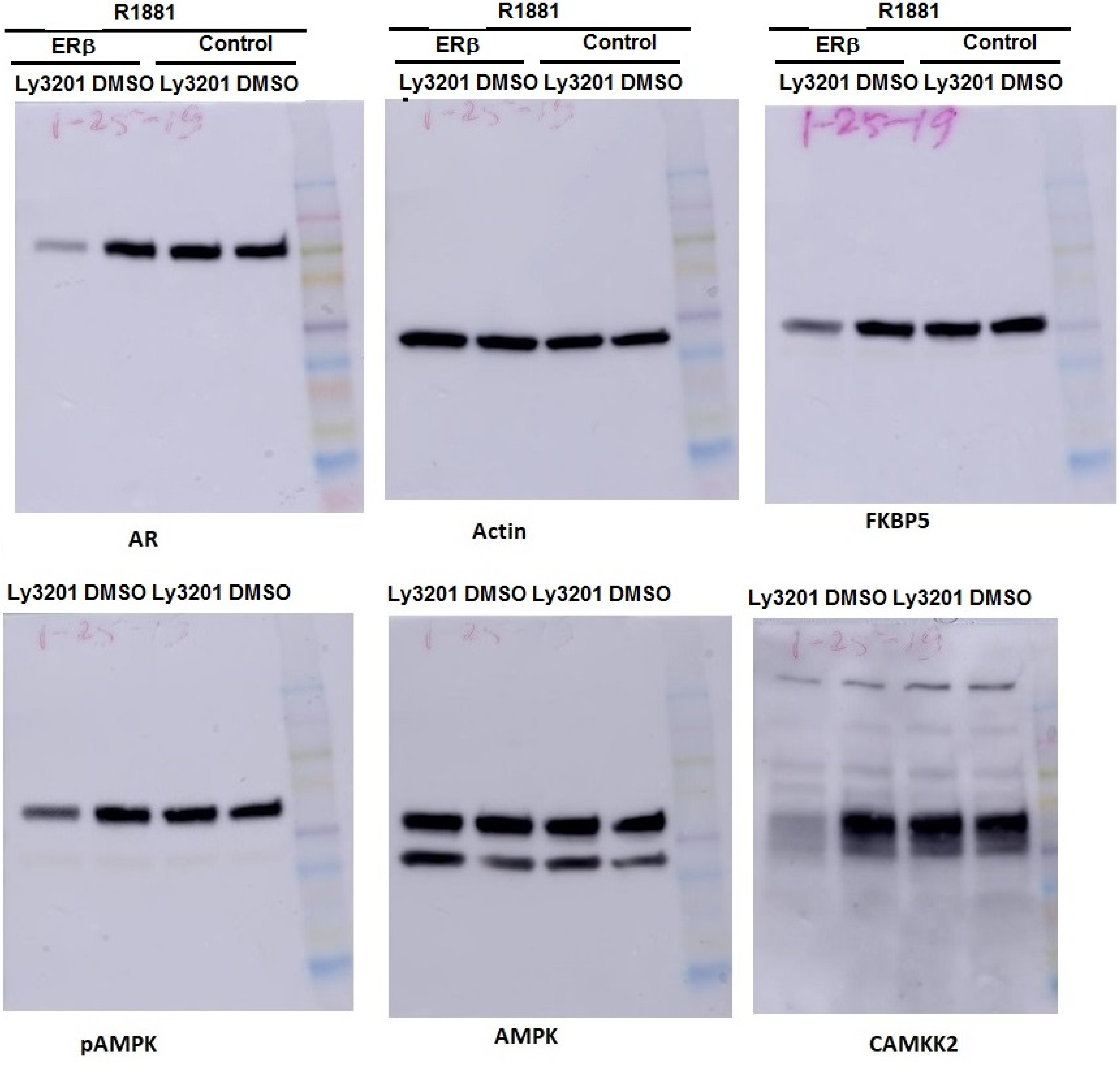
R1881
ERβ
Control
Ly3201 DMSO Ly3201 DMSO
AR
R1881
ERβ
Control
Ly3201 DMSO Ly3201 DMSO
Actin
R1881
ERβ
Control
Ly3201 DMSO Ly3201 DMSO
FKBP5
Ly3201 DMSO Ly3201 DMSO
pAMPK
Ly3201 DMSO Ly3201 DMSO
AMPK
Ly3201 DMSO Ly3201 DMSO
CAMKK2
